# Received

**Published:** 1870-02

**Authors:** 


RECEIVED.—We are happy to acknowledge the receipt of a very excellent steel engraved portrait of Dr. John W. Francis of New York, from the New York Medical and Psychological Journals, by whom it is dedicated to the United States.—It is of large size, and would adorn the handsomest apartment of a physicinn, and is given as a present to new subscribers to these journals.

